# LRG‐1 promotes fat graft survival through the RAB31‐mediated inhibition of hypoxia‐induced apoptosis

**DOI:** 10.1111/jcmm.17280

**Published:** 2022-03-23

**Authors:** Chia‐kang Ho, Danning Zheng, Jiaming Sun, Dongsheng Wen, Shan Wu, Li Yu, Ya Gao, Yifan Zhang, Qingfeng Li

**Affiliations:** ^1^ Department of Plastic & Reconstructive Surgery School of Medicine Shanghai Ninth People’s Hospital Shanghai Jiao Tong University Shanghai China

**Keywords:** apoptosis, autologous fat transplantation, fat graft survival, LRG‐1, RAB31

## Abstract

Autologous adipose tissue is an ideal soft tissue filling material, and its biocompatibility is better than that of artificial tissue substitutes, foreign bodies and heterogeneous materials. Although autologous fat transplantation has many advantages, the low retention rate of adipose tissue limits its clinical application. Here, we identified a secretory glycoprotein, leucine‐rich‐alpha‐2‐glycoprotein 1 (LRG‐1), that could promote fat graft survival through RAB31‐mediated inhibition of hypoxia‐induced apoptosis. We showed that LRG‐1 injection significantly increased the maintenance of fat volume and weight compared with the control. In addition, higher fat integrity, more viable adipocytes and fewer apoptotic cells were observed in the LRG‐1‐treated groups. Furthermore, we discovered that LRG‐1 could reduce the ADSC apoptosis induced by hypoxic conditions. The mechanism underlying the LRG‐1‐mediated suppression of the ADSC apoptosis induced by hypoxia was mediated by the upregulation of RAB31 expression. Using LRG‐1 for fat grafts may prove to be clinically successful for increasing the retention rate of transplanted fat.

## 1 INTRODUCTION

Congenital malformations and trauma often cause soft tissue defects, which can cause a series of physical and psychological problems, and have become common diseases that seriously endanger the physical and mental health of patients.^1^ At present, autologous tissues and synthetic materials are commonly used soft tissue filling materials. Because synthetic artificial materials have a series of shortcomings such as poor tissue compatibility, a clear sense of contours and nonpermanent implantation, these shortcomings hinder their widespread use.^2^ Among autologous tissues, autologous fat is the most common filling material, and fat transplantation is considered one of the best ways to repair defects.^3^, ^4^

Although autologous fat transplantation has many advantages such as abundant source, easy sampling and quick recovery, good histocompatibility and no foreign body rejection, resorption rates ranging from 25% to 80% have been reported, and the high and unpredictable absorption rate limits its clinical application.^5^ Studies suggest that reconstruction of microcirculation in the transplanted fat could promote fat graft survival by bringing more nutrients and oxygen to adipose stem cells.^6^, ^7^ Hence, scholars have attempted to add a variety of angiogenic molecules (Thymosin beta 4,^8^ VEGFA,^9^ ANG‐1 and IGF‐1^10^) to improve the survival rate of fat by improving the formation of blood vessels. However, neovascularization usually grows into the graft 5–7 days after transplantation, and neovascularization can only invade the periphery of the graft. Therefore, these methods have limited effects in improving the survival of transplanted fat and cannot be used in clinical applications. Before neovascularization develops, the graft must face a period of hypoxia, so other researchers thought that the resilience of adipocytes to hypoxia and relative macrophage activation play crucial roles in fat graft retention.^11^, ^12^, ^13^ Adipocytes are sensitive to stress and hypoxia, which are the two major obstacles in large‐volume fat grafting.^14^ Researchers found that apoptosis induced by many factors in the graft environment such as hypoxia and inflammation is a cause of long‐term volume reduction of the fat graft.^10^ A recent study suggests that adipose tissue from donors with high BMI demonstrates greater resistance to hypoxia‐induced apoptosis and has a higher survival rate.^12^ In summary, it is not difficult to find that the inhibition of fat cell apoptosis plays an important role in fat graft survival, whether from the perspective of promoting angiogenesis, balancing the immune microenvironment or resisting hypoxic damage.

The important role of ADSCs (adipose‐derived stem cells) in fat graft survival has been revealed in the ‘cell replacement theory’.^15^ It has shown convincing evidence of dynamic remodelling of adipose tissue after fat grafting that most adipocytes (except for those located superficially) die as early as day 1 after fat grafting, and the subsequent regeneration process generated by ADSCs starts.

LRG‐1, a highly conserved member of the leucine‐rich repeat family of proteins, has been reported to be involved in several pathophysiological processes. A study revealed that LRG‐1 could modulate pathological angiogenesis by directly binding to the TGF‐β accessory receptor endoglin.^16^ Researchers also found that LRG‐1 competes with apoptosis activating factor‐1 (Apaf‐1) to bind cytochrome c (Cyt c) and could play a role in the inhibition of apoptosis.^17^ Our previous research found that LRG‐1 can resist the damage caused by hypoxia, so it can normalize an abnormal process of diabetic wound healing where HIF‐1α stability is insufficient.^18^ A recent study has defined LRG‐1 as an adipokine secreted by adipocytes promoting de novo lipogenesis and the expression of LRG‐1 was significantly increased in obese people in previous data.^19^, ^20^ In general, LRG‐1 is closely related to promoting angiogenesis, resisting hypoxic damage, inhibiting apoptosis and promoting lipogenesis. Because we hope to improve these processes in fat transplantation, we are very curious about the possible role of LRG‐1 in fat transplantation and the specific mechanism.

In this study, we attempted to investigate whether LRG‐1 improves the survival rate of fat transplantation in a nude mouse model of fat grafts. Then, we tested its possible effect on h‐ADSC apoptosis cultured under hypoxia. Furthermore, we investigated the biochemical mechanism underlying the effects of LRG‐1 on graft survival.

## 2 METHODS

### 2.1 Sample acquisition

Adipose tissues were harvested from adult female patients (*n* = 8; mean age, 28 years; body mass index (BMI) range, 22.5–25.9 kg/m^2^; mean BMI, 24.3 kg/m^2^) during thigh liposuction in Shanghai Ninth People's Hospital with ethics approval from the local Human Research Ethics Committee of Shanghai Jiao Tong University School of Medicine in accordance with the principles of the Declaration of Helsinki. Written informed consent was obtained from patients undergoing surgery to obtain adipose tissue.

### 2.2 Animal ethics

Animal welfare strictly adhered to the principles of the ‘Guide for the Care and Use of Laboratory Animals’ (National Research Council. National Academies Press; 27 December 2010). All procedures were performed in accordance with the Guide for the Care and Use of Laboratory Animals, which was approved by the Committee on the Ethics of Animal Experiments of Shanghai Jiao Tong University School of Medicine. Room temperature was controlled by reheating units inside rooms and was maintained at 23 ± 2°C. The humidity was maintained at 30 to 70%. Animals were maintained on a 12:12‐h light:dark cycle (lights on, 8 a.m. to 8 p.m.). At the end of the in vivo experiment, euthanasia was conducted according to ‘CCAC guidelines on euthanasia of animals used in science. Canadian Council on Animal Care’.

### 2.3 Fat graft model

In the first group of animal experiments, mice were randomly divided into three groups (16 mice per group): PBS group, 1 μg/ml LRG‐1 group and 5 μg/ml LRG‐1 group. Each mouse was injected subcutaneously on the left and right flank of the back with 0.3 ml of Coleman fat. The mice were locally injected with 0.1 ml of PBS on the left and 1 μg/ml or 5 μg/ml LRG1 (7890‐LR‐025, R&D Systems) on the right according to different groups every other day. At 2 weeks, fat graft apoptosis tests were conducted in different groups of mice (8 mice per group). At 12 weeks, the remaining mice were scanned with microCT, the grafts were harvested, and their volumes and weights were measured. Each harvested sample was assessed histologically and immunohistochemically.

In the second group of animal experiments, the Coleman fat injected was randomly divided into four groups (16 Coleman fat per group): control group (fat and PBS injection), LRG‐1 group (fat and 5 μg/ml LRG‐1 injection), AAV‐shCtrl group (fat and 5 μg/ml LRG‐1 and AAV‐shCtrl injection) and AAV‐shRAB31 group (fat and 5 μg/ml LRG‐1 and AAV‐shRAB31 injection).

### 2.4 MicroCT analysis

The fat grafts were scanned with microCT (PerkinElmer, USA), and the results were analysed by ProPlan CMF 3.0.

### 2.5 Human adipose‐derived stem cell isolation and cell culture

The collected fat particles were washed with phosphate‐buffered saline (PBS) and incubated for 5 min at room temperature. The lower layer of tumescent fluid and blood components were discarded. Then, the fat particles were mixed with an equal volume of prepared type IV collagenase solution (Gibco, USA). The solution was dissolved in low‐glucose Dulbecco's modified Eagle's medium (low‐glucose DMEM, HyClone, USA) at a final concentration of 0.2% and filtered twice with a 0.22‐μm filter (Falcon, USA). The cells were shaken on a 37°C shaker at 4 ×*g* for 1 h. After shaking, the solution was centrifuged at 239‐425 ×*g* for 5 min, and the supernatant and fat suspension were discarded. Ten millilitres of low‐glucose DMEM with 10% foetal bovine serum (FBS, Gibco, USA) and 1% antibiotic‐antimycotic (Gibco, USA) was added to the centrifuge tube (Falcon, USA), pipetted evenly and centrifuged at 106 ×*g* for 5 min. The supernatant was discarded, and cell pellets were seen at the bottom of the tube. Three millilitres of FBS‐containing medium was added and pipetted evenly; then, 8 ml of serum‐containing medium was added, mixed well and inoculated in a 10‐cm Petri dish. For the normoxic environment, cells were cultured in triplicate under normoxic conditions (21% O_2_). For the hypoxic environment, cells were further cultured in triplicate under hypoxic conditions (1% O_2_).

### 2.6 Histology and immunohistochemistry

Tissues that were paraformaldehyde‐fixed overnight and then paraffin‐embedded were cut at a thickness of 5 μm and then stained with haematoxylin and eosin (H&E) following our previous study.^21^ For immunohistochemistry staining, sections were incubated with primary antibody against F4/80 (Abcam, ab60343, 1:100) or cleaved caspase‐3 (Abcam, ab32042, 1:200) diluted in blocking solution overnight at 4 °C. After incubation with HRP‐conjugated secondary antibody, the sections were counterstained with haematoxylin and developed with diaminobenzidine.

### 2.7 Immunofluorescence cell staining

For immunofluorescent staining, tissue sections were incubated with primary antibody against Perilipin (Abcam, ab172907, 1:200) diluted in blocking solution overnight at 4°C. After incubation with Alexa Fluor 488–conjugated goat antirabbit immunoglobulin G (Invitrogen, USA, 1:500), nuclei were stained with 4′,6‐diamidino‐2‐phenylindole (SouthernBiotech, USA). Image‐Pro Plus 6.0 software was used for quantitative analysis.

### 2.8 Apoptosis assay

For cell Annexin V and PI staining, 195 μl of cell suspension was mixed well with 5 μl Annexin V‐FITC (Invitrogen, BMS500FI‐20) and incubated at room temperature for 10 min. Cells were washed with PBS and resuspended in 190 μl deliquated binding buffer, and then, 10 μl PI (20 µg/ml) was added. The samples were analysed by flow cytometry using the Cell Quest program (BD Biosciences, San Jose, CA, USA).

For fat graft Annexin V and PI staining, a previous study was referenced.^22^ Briefly, grafts were washed with PBS and placed on ice. Grafts were digested with 1 mg/ml collagenase buffer and incubated at 37°C for 30 min with vigorous shaking. After digestion, EDTA was added to a final concentration of 10 mM and incubated at 37 °C for an additional 5 min. The next steps were as described for Annexin V and PI staining.

### 2.9 RNA purification and quantitative real‐time PCR (RT‐qPCR)

Total RNA was isolated using TRIzol reagent (Invitrogen). RT‐qPCR was performed with an ABI 7900HT system using SYBR Premix (Takara, Dalian, China) according to the manufacturer's instructions. Glyceraldehyde‐3‐phosphate dehydrogenase (GAPDH) was used as an internal control. The primers used in this study were as follows: GAPDH: forward, 5′‐GGAGCGAGATCCCTCCAAAAT‐3′; reverse, 5′‐GGCTGTTGTCATACTTCTCATGG‐3′; RAB31: forward, 5′‐GGGGTTGGGAAATCAAGCATC‐3′; reverse, 5′‐GCCAATGAATGAAACCGTTCCT‐3′.

### 2.10 Western blotting

Tissues and cultured cells were lysed with RIPA buffer supplied with protease inhibitor cocktail (Roche, Mannheim, Germany). Concentrations of protein were detected by the bicinchoninic acid (BCA) assay (Thermo Fisher Scientific). To analyse inducible protein expression, 20 μg protein was resolved by 10% or 12% sodium dodecyl sulphate‐polyacrylamide gel electrophoresis (SDS‐PAGE) and electroblotted in polyvinylidene difluoride (PVDF) membranes (Millipore, Bedford, MA, USA). The membranes were blocked with 5% nonfat milk at room temperature for 1 h. The separated proteins were then immunoblotted and probed with primary anti‐Bax antibody (Abcam, ab32503, 1:5000), antiBcl‐2 antibody (Abcam, ab182858, 1:2000), anti‐Cleaved caspase 3 antibody (Abcam, ab208003, 1:1000), antiRAB31 antibody (Abcam, ab230881, 1:1000), antiβ‐actin antibody (Abcam, ab8227, 1:5000), antiMLKL (Abcam, ab184718, 1:1000), antip‐MLKL (Abcam, ab196436, 1:1000), antiRIPK1 (CST, 3493T, 1:1000), antip‐RIPK1 (proteintech, 66854‐1‐Ig, 1:500), antiRIPK3 (proteintech, 17563–1‐AP, 1:500), antip‐RIPK3 (Abcam, ab184718, 1:500), antiLc3b (Abcam, ab192890, 1:1000), antip62 (Abcam, ab109012, 1:1000), antiATGL (Abcam, ab207799, 1:1000) and antiHSL (Abcam, ab109400, 1:50000) at 4°C overnight. The next day, the membranes were incubated with peroxidase‐conjugated secondary antibody (1:10000) (Nebraska, USA) at room temperature for 1 h after washing with TBST three times for 5 min. ImageJ software was used for quantitative analysis, which was conducted on immunoreactive bands.

### 2.11 5‐ethynyl‐2’‐deoxyuridine (EdU) proliferation assay

Cells were seeded in 24‐well plates incubated with different concentrations of rhLRG‐1 (0, 300 ng/ml) under normoxic or hypoxia conditions. Twenty‐four hours after incubation, cell proliferation was detected using the EdU Cell Proliferation Assay Kit (Invitrogen, USA) according to the manufacturer's protocol. Briefly, cells were incubated with 50 mM EdU for 2 h before fixation, permeabilization and EdU staining. Then, cell nuclei were stained with DAPI (Sigma‐Aldrich, St. Louis, MO) at a concentration of 1 mg/ml for 8 min. The proportion of cells that incorporated EdU was determined by Zeiss 710 laser‐scanning microscope (Zeiss, Oberkochen, Germany).

### 2.12 Biochemical analysis

Plasma‐free fatty acids were quantified using a specific assay kit from Abcam (Abcam, ab65341). Plasma triglyceride were quantified using a specific assay kit from Abcam (Abcam, ab65336). For detailed operation steps, please refer to the corresponding manual.

### 2.13 siRNA and plasmid transfection

For RAB31 silencing, h‐ADSC were transfected in 6‐well plates with 100 nM (final) RAB31 siRNA (SR307545, OriGene Technologies) using Lipofectamine RNAiMAX reagent (Invitrogen, Carlsbad, CA, USA) according to the manufacturer's protocol. Nontargeting (NT) siRNA (sc‐37007) was used as a negative control.

### 2.14 AAV vector administration

We utilized the AAV Helper‐Free System (AAV Helper‐Free System, Stratagene) for viral production using a triple‐transfection, helper‐free method and purified it as described in a previous study.^23^ The interference sequences were as follows: shRNA: 5′‐CCGGTTATGTGTATGGGATTCTAAACTCGAGTTTAGAATCCCATACACATAATTTTTG‐3′; and control shRNA, 5′‐TTCTCCGAACGTGTCACGT‐3′. Briefly, nude mice were anaesthetized with an isoflurane/air mix (3% for initial induction and 1.5–2% for maintenance). Three hundred nanolitres of either AAV5‐shRAB31 or AAV5‐shCtrl was injected into the Coleman fat with LRG‐1 according to different groups as mentioned before. The injections were performed using a 34‐gauge needle (World Precision Instruments) attached to a 10‐μl NanoFil microsyringe (Nanofil, World Precision Instruments).

### 2.15 Statistical analysis

Each experiment was performed at least three times. The data were analysed with the statistical software package SPSS 20.0 (SPSS, Chicago, IL, USA). The Mann−Whitney nonparametric test or Student's *t*‐test was used during analysis. Differences in gene expression in the public dataset were calculated using the Kruskal–Wallis test. The difference between groups was regarded as considerable at *p* < 0.05.

## 3 RESULTS

### 3.1 LRG‐1 facilitated the survival rate of transplanted fat

**FIGURE 1:**
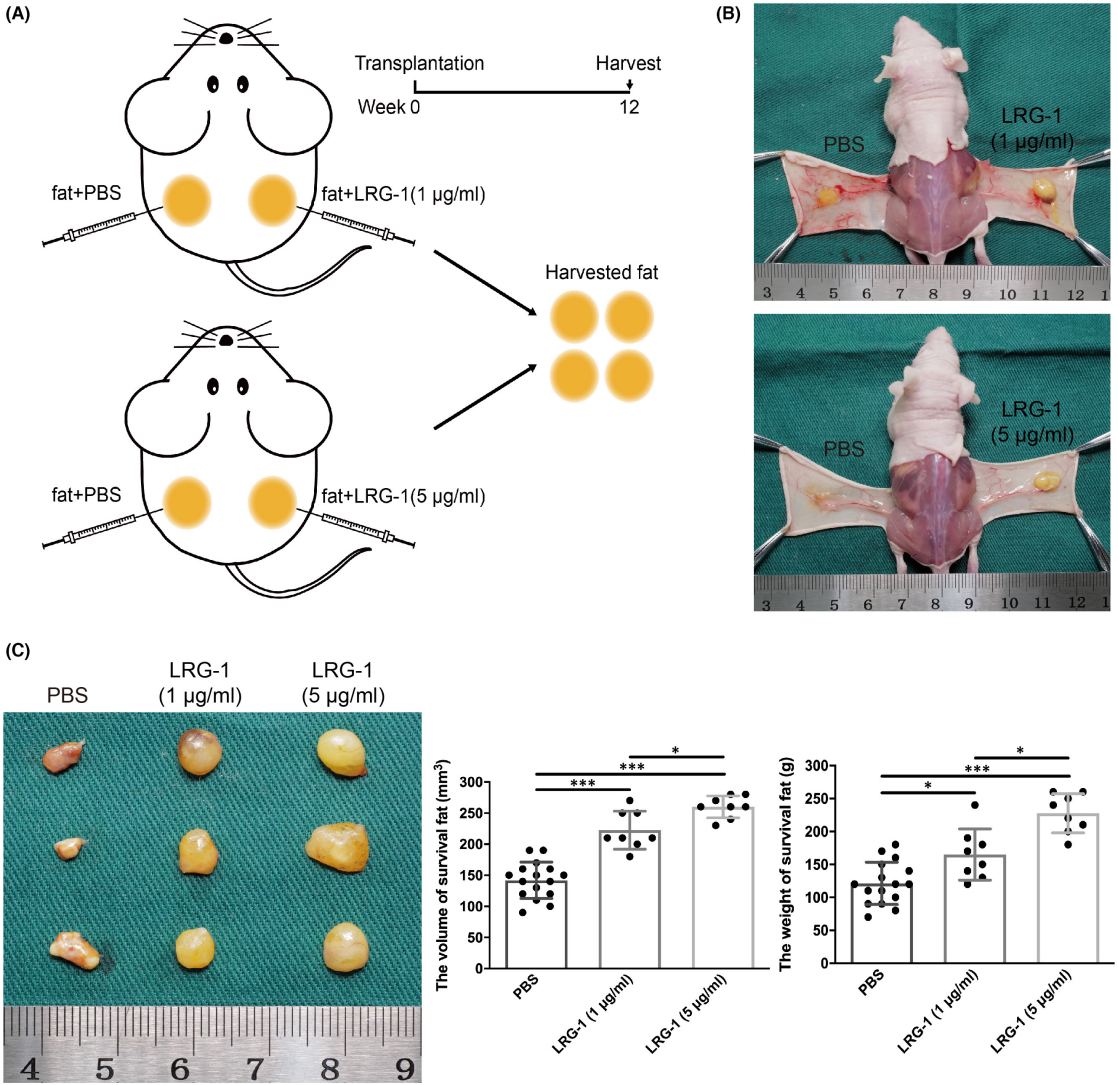
LRG‐1 facilitated the long‐term survival of transplanted fat. (A) Schematic diagram of the nude mouse Coleman fat graft model experimental design and schedule. (B) The mouse was injected subcutaneously into the left and right flanks of the back. (C) Representative image of macroscopic views of harvested grafts in the control and LRG‐1‐treated groups. (D) Measurements of the volume and weight of fat grafts in different groups. Data are presented as the mean ± SD. (*n* = 8 biologically independent animals) *p < 0.05, ***p < 0.001

**FIGURE 2:**
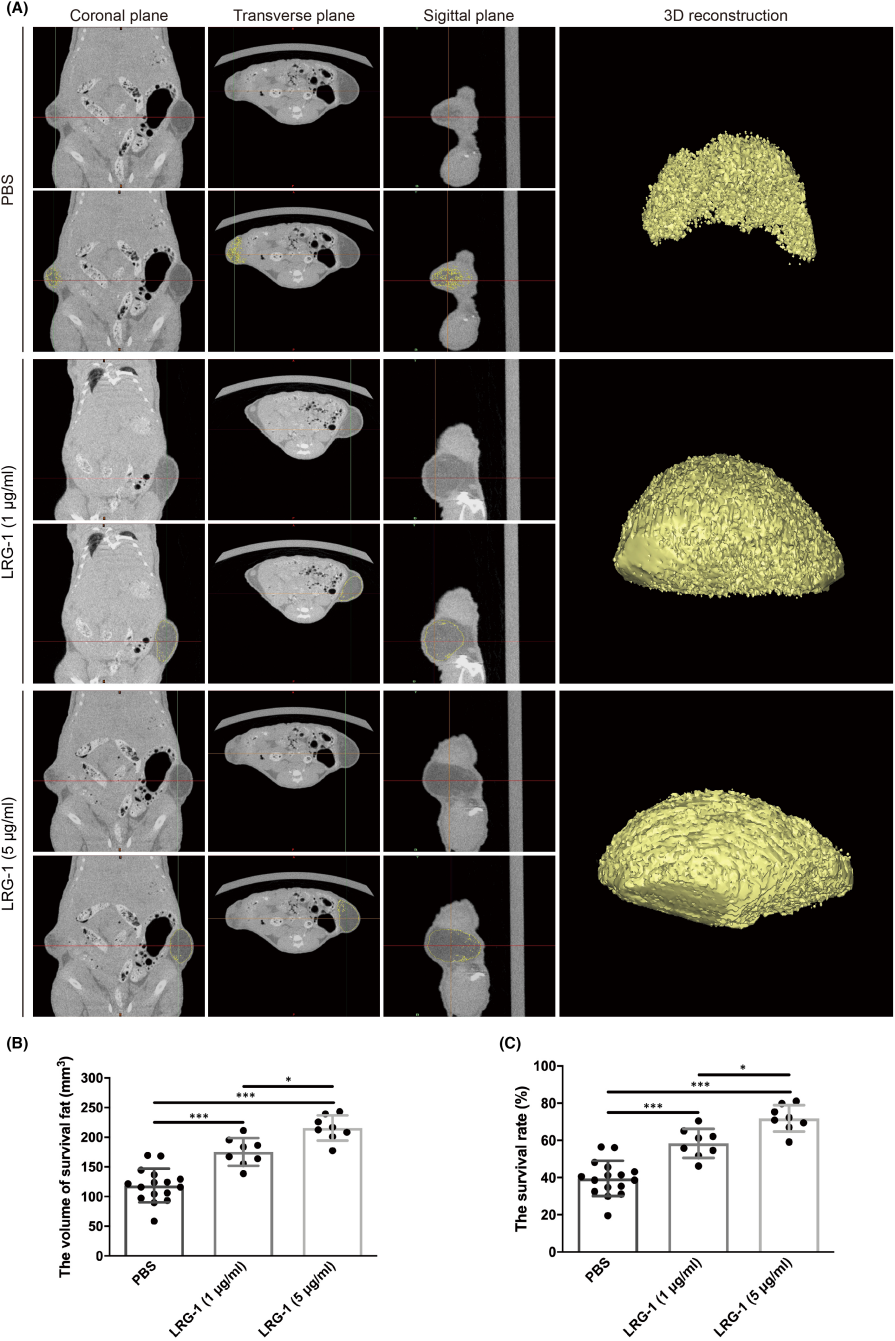
Analysis of fat survival in a nude mouse fat graft model at Week 12 by microCT. (A) MicroCT analysis of the subcutaneous transplantation in three sections (sagittal section, transverse section and coronal section), where yellow represents surviving fat. (B, C) Measurement of the volume of surviving fat and survival rate by ProPlan CMF 3.0. Data are presented as the mean ± SD. (*n* = 8 biologically independent animals) *p < 0.05, ***p < 0.001

First, the role of the LRG‐1 protein in fat transplantation was investigated using a nude mouse fat transplantation model (Figure 1A, B). Then, the volume and weight of the transplanted fat in different groups were measured at 12 weeks and 1 year. Statistics demonstrated that fat grafts were heavier and larger with either injection of 1 μg/ml or 5 μg/ml LRG‐1 than in the control group at 12 weeks (Figure 1C). In addition, compared with the control group, the fat grafts in the LRG‐1 injection group still had greater volume and less calcification and oil cyst after one year (Figure S1A, B). Tissue HE staining also showed that the fat grafts in the LRG‐1 injection group still maintained better integrity and less calcification and oil cyst (Figure S1C). These results indicated that LRG‐1 could promote the survival of fat grafts. To observe the survival of fat transplantation more intuitively, a microCT scan was carried out. The 3D reconstruction of microCT suggested that the two LRG‐1 injection groups, either 1 μg/ml or 5 μg/ml, showed larger survival volumes than the control group; representative images are shown in Figure 2A. The statistical results showed that the volume and survival rate of fat grafts were significantly higher in the two groups injected with LRG‐1 (Figure 2B, C). In addition, 5 μg/ml LRG‐1 injection could assist fat graft survival by more than 1 μg/ml LRG‐1. In general, LRG‐1 facilitated the survival rate of fat grafts.

### 3.2 LRG‐1 mitigated the apoptosis of the transplanted fat

**FIGURE 3:**
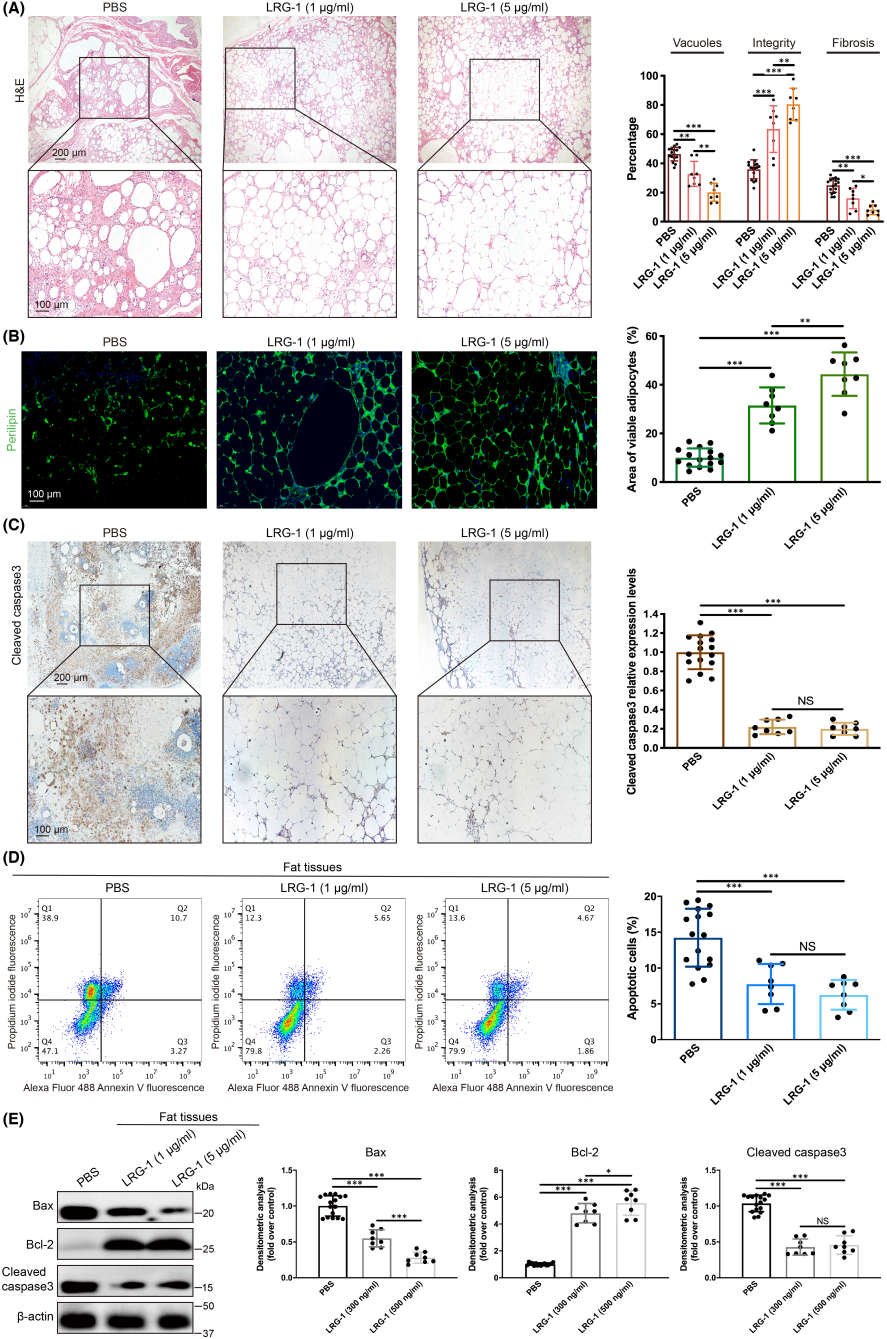
LRG‐1 mitigated the apoptosis of the transplanted fat. (A) Images of H&E‐stained sections of the PBS injection and LRG‐1 injection groups. (Scale bar = 200/100 μm). (B) Images and quantitative analysis of immunofluorescence staining of Perilipin (green). (DAPI (blue) for nuclei staining, Scale bar  = 100 μm). (C) Images and quantitative analysis of immunohistochemistry staining of cleaved caspase‐3. (Scale bar = 200/100 μm). (D) Apoptosis was detected in fat grafts in the PBS‐ and LRG‐1‐injected groups by flow cytometry. (E) Western blot analysis of Bax, Bcl‐2 and cleaved caspase‐3 in fat grafts in different groups. Data are presented as the mean ± SD. (*n* = 8 biologically independent animals) *p < 0.05, **p < 0.01, ***p < 0.001

To explore the specific mechanism by which LRG‐1 promotes the survival of transplanted fat, the histology of fat grafts in different groups was investigated. Statistical analyses of fat integrity, vacuoles and fibrosis confirmed that higher fat integrity, fewer vacuoles and less fibrosis were observed in the LRG‐1 injection groups than in the control group (Figure 3A). Furthermore, our immunofluorescence assay showed that the number of Perilipin + living adipocytes in the LRG‐1‐treated groups was significantly higher than the number of Perilipin + living adipocytes in the control group (Figure 3B). In addition, by immunohistochemical staining, we found that the F4/80^+^ macrophage infiltration level of the LRG‐1‐treated groups was less than the F4/80^+^ macrophage infiltration level of the control group (Figure S2). As previous studies have shown that increased macrophage infiltration is closely related to increased apoptosis in adipose tissue,^24^, ^25^ we tested apoptosis in transplanted fat tissue. The immunohistochemical results of cleaved caspase‐3 showed that LRG‐1 injection significantly reduced apoptosis in fat grafts (Figure 3C). Similarly, the flow cytometry results demonstrated that LRG‐1 injection markedly reduced the apoptosis of fat grafts to a large extent (Figure 3D). Moreover, LRG‐1 injection inhibited the expression of proapoptotic proteins (Bax and cleaved caspase‐3) and promoted the expression of antiapoptotic proteins (Bcl‐2) in fat grafts (Figure 3E). From the above in vivo experiments, it is not difficult to find that LRG‐1 could inhibit the apoptosis of transplanted fat.

### 3.3 LRG‐1 could reduce hypoxia‐induced h‐ADSC apoptosis

**FIGURE 4:**
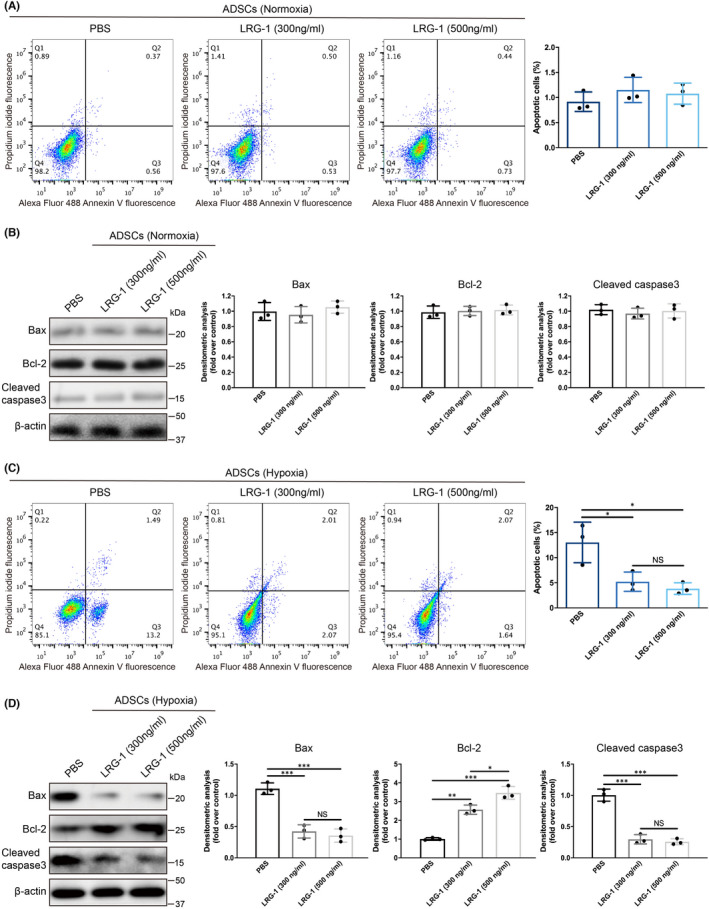
LRG‐1 reduced hypoxia‐induced h‐ADSC apoptosis. (A) Apoptosis was detected after treating h‐ADSC with PBS or LRG‐1 for 48 h under normal oxygen (21% O_2)_ culture conditions by flow cytometry. (B) Western blot analysis of Bax, Bcl‐2 and cleaved caspase‐3 in h‐ADSC after treatment with PBS or LRG‐1 (300 ng/ml or 500 ng/ml) for 48 h under normal oxygen (21% O_2_). (C) Apoptosis was detected after treating h‐ADSC with PBS or LRG‐1 for 48 h under hypoxic (1% O_2_) culture conditions by flow cytometry. (D) Western blot analysis of Bax, Bcl‐2 and cleaved caspase‐3 in h‐ADSC after treatment with PBS or LRG‐1 for 48 h under hypoxia (1% O_2_). Data are presented as the mean ± SEM. (*n* = 3 independent experiments) *p < 0.05, **p < 0.01, ***p < 0.001

To further understand the inhibitory effect of LRG‐1 on adipocyte apoptosis, in vitro experiments related to apoptosis were conducted. After the addition of exogenous LRG‐1 (300 or 500 ng/ml) to the culture medium of human adipose‐derived stem cell (h‐ADSC), flow cytometry was used to measure cell apoptosis. The results showed that under normal oxygen (21% O_2_) culture conditions, h‐ADSC rarely underwent apoptosis, so LRG‐1 had no significant effect on their apoptosis (Figure 4A, B). Previous studies have shown that transplanted fat grafts are in a state of hypoxia.^26^ Therefore, the effect of LRG‐1 on the apoptosis of h‐ADSC cultured under hypoxia (1% O_2_) was investigated. The results showed that LRG‐1 addition reduced hypoxia‐induced h‐ADSC apoptosis to a large extent (Figure 4C). Moreover, the protein expression levels of Bax and cleaved caspase‐3 were increased, while Bcl‐2 was decreased in h‐ADSC cultured under hypoxia (Figure S4A). The addition of LRG‐1 inhibited the expression of proapoptotic proteins (Bax and cleaved caspase‐3) induced by hypoxia and promoted the expression of antiapoptotic proteins (Bcl‐2) inhibited by hypoxia (Figure 4D). However, Western blot results showed that LRG‐1 had no significant effect on the expression of proteins related to other types of cell death including autophagy and necroptosis (Figure S4A–E) and EdU proliferation assay demonstrated that LRG‐1 addition had no significant effect on h‐ADSC proliferation (Figure S4F, G). Taken together, these results suggested that exogenous LRG‐1 could regulate the expression of apoptosis‐related proteins and inhibit hypoxia‐induced h‐ADSC apoptosis.

### 3.4 **RAB31** **mediated the inhibitory effect of LRG‐1 on hypoxia‐induced apoptosis in h‐ADSC**

**FIGURE 5:**
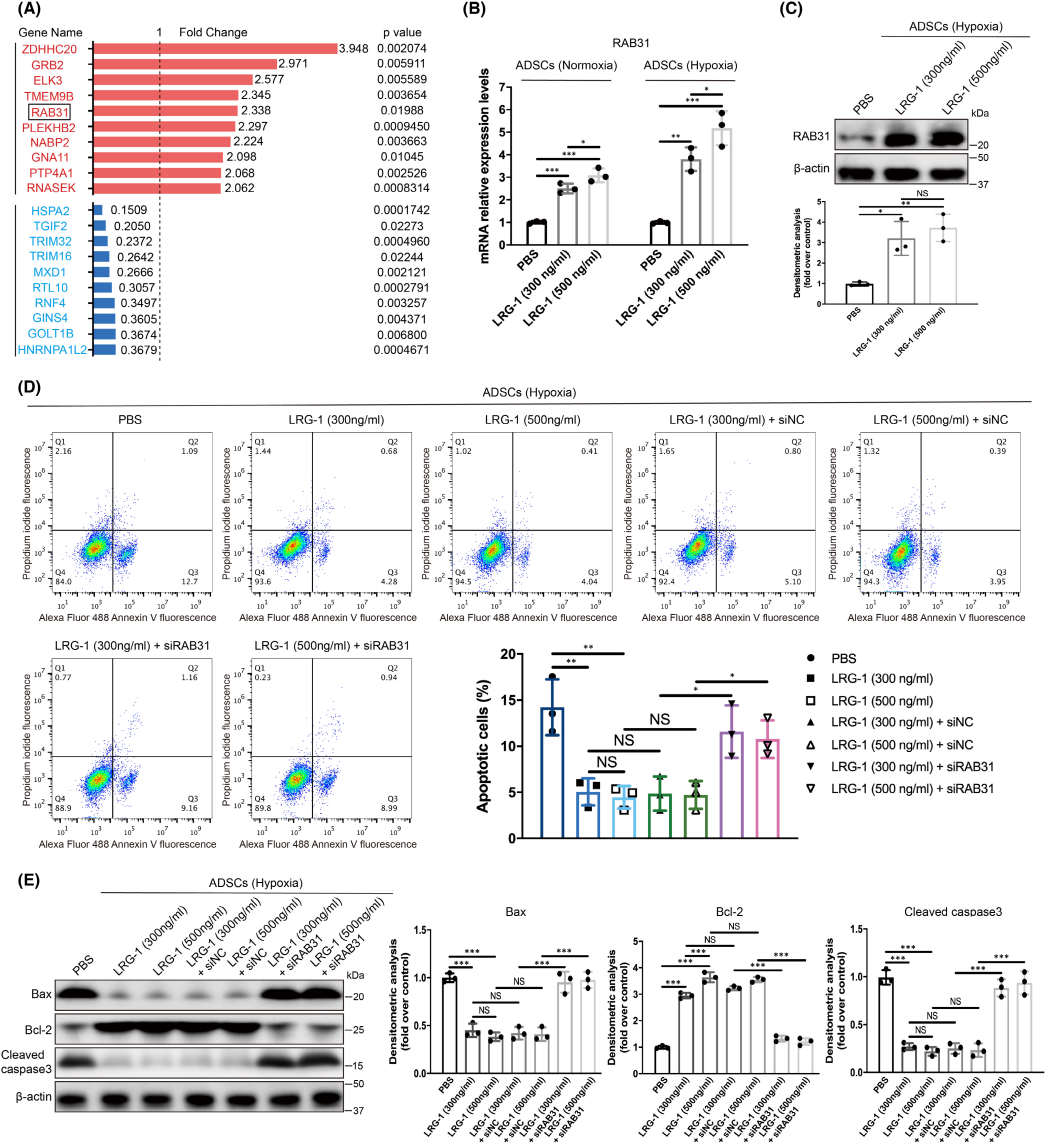
RAB31 mediated the inhibitory effect of LRG‐1 on apoptosis in h‐ADSC under hypoxic conditions. (A) Top 10 upregulated and downregulated genes from transcriptome data analysis of the control group and LRG‐1 additional group (GSE122527). (B) The levels of RAB31 mRNA in h‐ADSC treated with PBS or LRG‐1 for 48 h under normal oxygen (21% O2) or hypoxia (1% O2). (C) The levels of RAB31 protein in h‐ADSC treated with PBS or LRG‐1 for 48 h under hypoxia (1% O2). (D) Apoptosis was detected after treating h‐ADSC with PBS, LRG‐1, LRG‐1 with siNC and LRG‐1 with siRAB31 for 48 h under hypoxia (1% O2). (E) The protein levels of Bax, Bcl‐2 and cleaved caspase‐3 were detected in the different groups. Data are presented as the mean ± SEM. (*n* = 3 independent experiments) *p < 0.05, **p < 0.01, ***p < 0.001

To understand the specific mechanism by which LRG‐1 regulated h‐ADSC apoptosis, we compared the high‐throughput RNA sequencing gene expression profiles (GSE122527) conducted in our previous study. As shown in Figure 5A, the top 10 genes upregulated or downregulated in keratinocytes by LRG‐1 addition were subjected to searches in the GEO database to find a correlation with cell apoptosis. Among these genes, RAB31 drew our attention. First, through the detection of RT‐qPCR, we confirmed that the addition of LRG‐1 to h‐ADSC increased the mRNA expression level of RAB31 regardless of hypoxia or normoxia (Figure 5B). We found that hypoxia did not induce the expression of RAB31 (Figure S5A), while LRG‐1 addition increased the protein expression of RAB31 in h‐ADSC under hypoxia (Figure 5C). As a literature review showed that RAB31 is closely related to apoptosis,^27^, ^28^ we implemented siRNA experiments against RAB31 to inhibit its expression prior to hypoxic culture (Figure S5B). The results showed that RAB31 knockdown significantly inhibited LRG‐1 addition reduced h‐ADSC apoptosis under hypoxia (Figure 5D). In addition, the results suggested that RAB31 knockdown relieved the addition of LRG‐1 by promoting the expression of Bax and cleaved caspase‐3 while inhibiting the expression of Bcl‐2 under hypoxia (Figure 5E). From the above results, it was not difficult to conclude that RAB31 mediated the inhibitory effect of LRG‐1 on h‐ADSC apoptosis under hypoxic conditions.

### 3.5 Knockdown of RAB31 inhibited the promoting effect of LRG‐1 on the survival of transplanted fat

**FIGURE 6:**
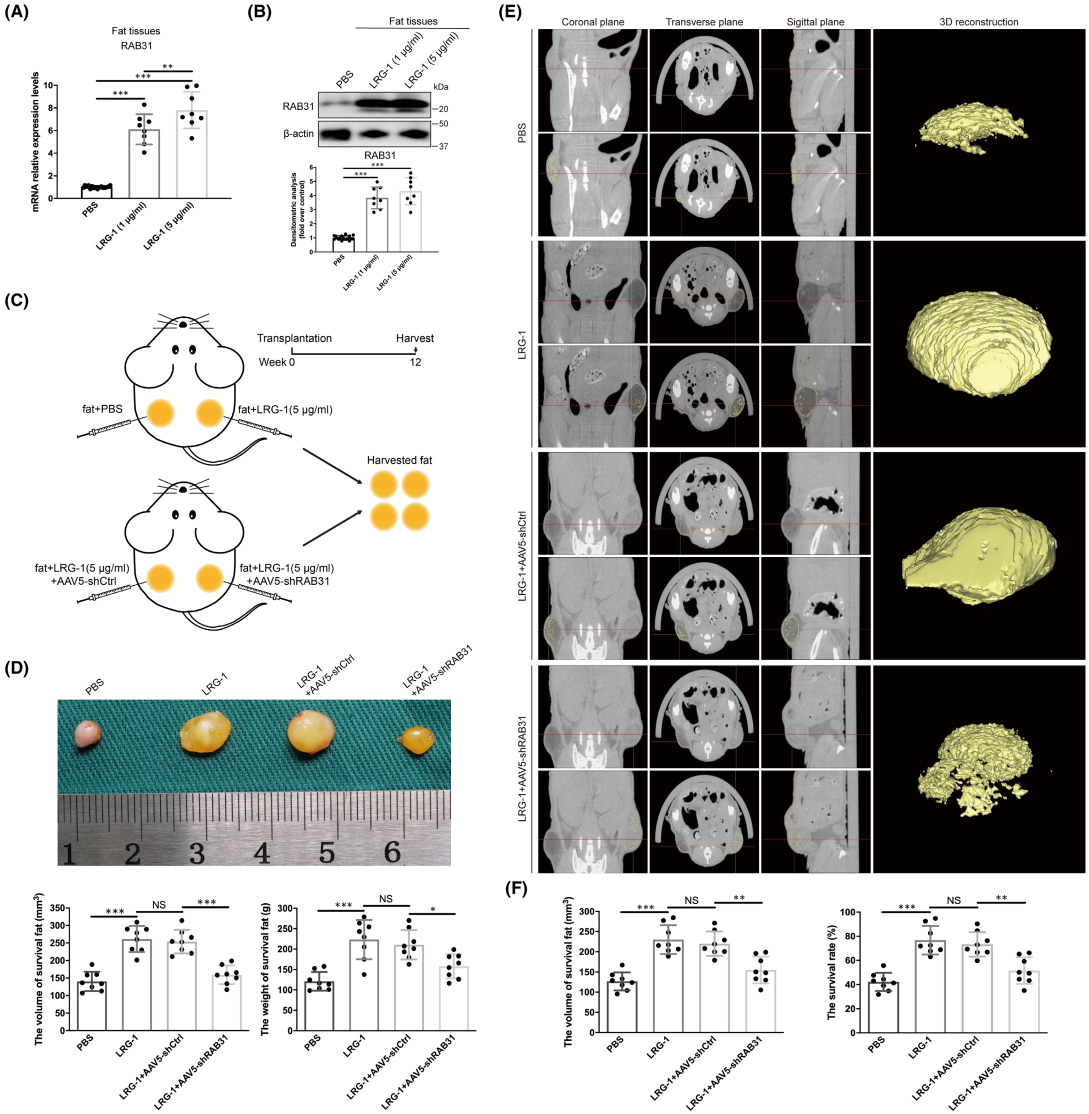
Knockdown of RAB31 inhibited the promoting effect of LRG‐1 on the survival of transplanted fat. (A, B) The levels of RAB31 mRNA and protein in the control and LRG‐1 injection groups at Week 12. (C) Schematic diagram of the nude mouse Coleman fat graft model experimental design and schedule. (D) Representative image of macroscopic views of harvested grafts in the control, LRG‐1 injection, LRG‐1 combined with AAV‐shCtrl injection and LRG‐1 combined with AAV‐shRAB31 injection groups. (E) MicroCT analysis of the subcutaneous transplantation in three sections (sagittal section, transverse section and coronal section); yellow represents surviving fat. (F) Measurement of the volume of surviving fat and survival rate by ProPlan CMF 3.0. Data are presented as the mean ± SD. (*n* = 8 biologically independent animals) *p < 0.05, **p < 0.01, ***p < 0.001

To further confirm that LRG‐1 inhibited cell apoptosis through RAB31, we conducted in vivo experiments. The results showed that the injection of LRG‐1 increased both the mRNA and protein expression levels of RAB31 in the transplanted fat (Figure 6A, B). When fat grafts were treated with AAV5‐shRAB31, the expression of RAB31 was significantly downregulated compared with AAV5‐shCtrl‐treated fat grafts (Figure S6). We also investigated the role of RAB31 in fat transplantation (Figure 6C). The volume and weight quantification of the transplanted fat demonstrated that the fat grafts were smaller and lighter in the LRG‐1 combined with AAV‐shRAB31 injection group than in the LRG‐1 injection and LRG‐1 combined with AAV‐shCtrl injection group (Figure 6D). These results indicated that knockdown of RAB31 damaged the promotion effect of LRG‐1 on the survival of fat grafts. The 3D reconstruction of microCT and transplanted fat volume and weight quantification also confirmed this conclusion (Figure 6E, F).

**FIGURE 7:**
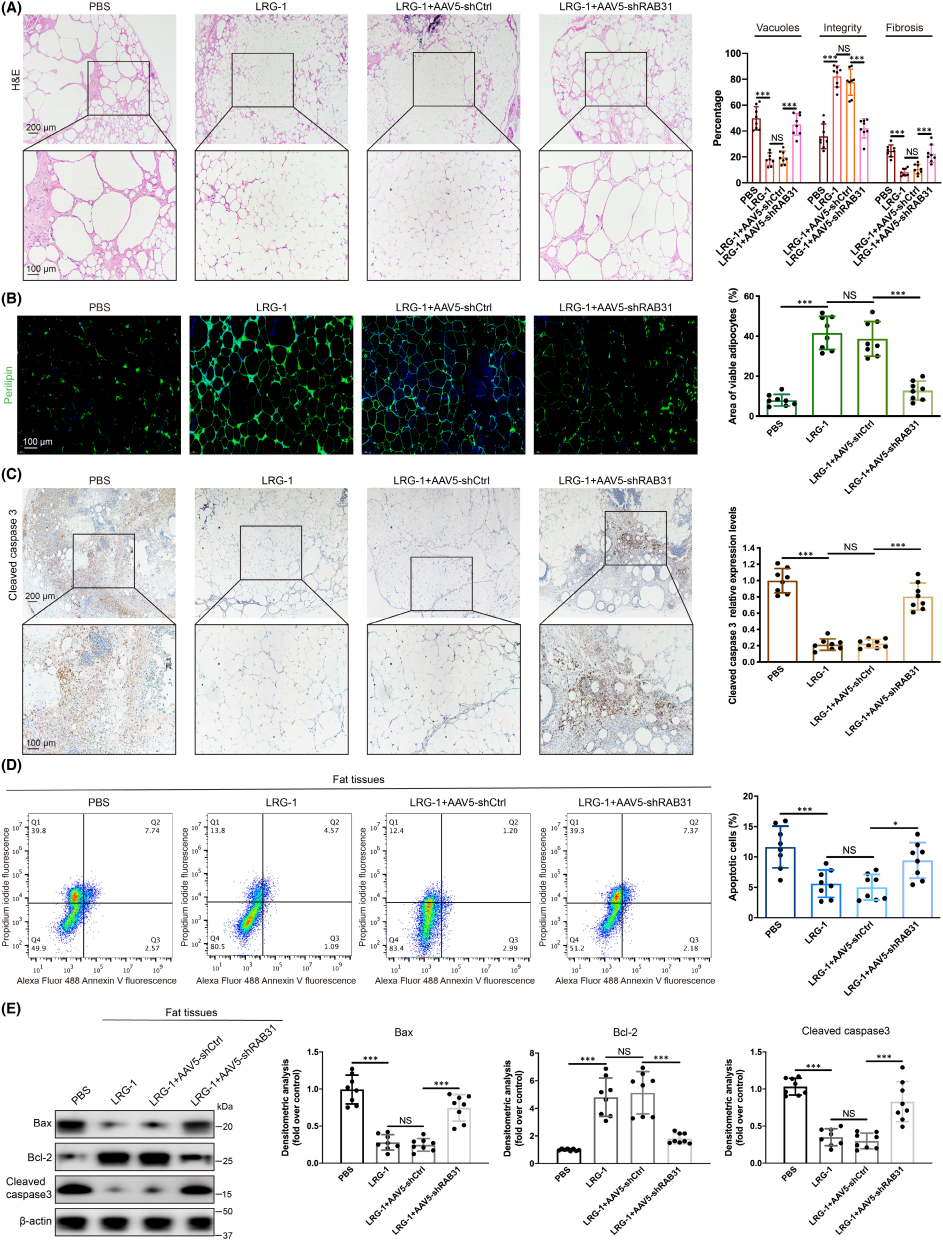
RAB31 mediated the inhibitory effect of LRG‐1 on apoptosis of fat grafts. (A) Images of H&E‐stained sections in the control, LRG‐1 injection, LRG‐1 combined with AAV‐shCtrl injection and LRG‐1 combined with AAV‐shRAB31 injection groups (scale bar = 200/100 μm). (B) Images and quantitative analysis of immunofluorescence staining of Perilipin (green). (DAPI (blue) for nuclei staining, Scale bar   =100 μm). (C) Images and quantitative analysis of immunohistochemistry staining of cleaved caspase‐3. (Scale bar = 200/100 μm). (D) Apoptosis was detected in fat grafts in the control, LRG‐1 injection, LRG‐1 combined with AAV‐shCtrl injection and LRG‐1 combined with AAV‐shRAB31 injection groups by flow cytometry. (E) Western blot analysis of Bax, Bcl‐2 and cleaved caspase‐3 in fat grafts in different groups. Data are presented as the mean ± SD. (*n* = 8 biologically independent animals) *p < 0.05, ***p < 0.001

In addition, we investigated the histology of fat grafts in different groups. When RAB31 was knocked down by shRNA, the positive effect of LRG‐1 on increasing fat integrity and decreasing vacuoles and fibrosis was obviously abolished (Figure 7A). Additionally, the number of Perilipin + living adipocytes was lower in the LRG‐1 combined with AAV‐shRAB31 injection group than in the LRG‐1 injection and LRG‐1 combined with AAV‐shCtrl injection group (Figure 7B). Furthermore, the immunohistochemistry assay of cleaved caspase‐3 showed that the AAV‐shRAB31 injection substantially inhibited the apoptosis inhibitory effect of LRG‐1 on transplanted fat (Figure 7C). Flow cytometry apoptosis analysis demonstrated similar results (Figure 7D). In addition, the Western blotting results showed that RAB31 knockdown reduced the regulatory effect of LRG‐1 on apoptosis‐associated proteins (Figure 7E). These in vivo results indicated that RAB31 mediated the apoptosis inhibitory effect of LRG‐1 on fat grafts.

## 4 DISCUSSION

Autologous adipose tissue is an ideal soft tissue filling material, and its biocompatibility is better than the biocompatibility of artificial tissue substitutes, foreign bodies and heterogeneous materials.^4^, ^29^ According to a survey, most surgeons have used fat transplantation in the clinic because of its many advantages such as abundant sources, easy sampling, quick recovery and no foreign body rejection.^30^ However, autologous fat transplantation faces the problems of a high postoperative absorption rate and low survival rate, which limit its wide clinical application.^29^ In the present study, we found that LRG‐1 injection could promote fat graft survival to a large extent in a nude mouse fat graft model, which may prove clinically successful in raising the retention rate of transplanted fat.

Several studies have shown that fat grafts initially lack vascular support and receive oxygen and nutrients only via diffusion until neovascularization develops.^26^ Cell death and apoptosis caused by ischaemia and hypoxia are important reasons for the low survival rate of transplanted fat.^15^, ^31^ Researchers found that hypoxia could induce cardiomyocyte apoptosis by targeting Yap1.^32^ A study also demonstrated that hypoxia‐induced HIF‐1α significantly induced apoptosis through both the cell death receptor and mitochondrial‐associated apoptosis pathways.^33^ During fat transplantation, researchers found that only peripheral adipocytes survived, as in the early stage after fat grafting, adipocytes obtain nutrients and oxygen through plasmatic diffusion from the surrounding tissues.^15^, ^34^ Given that inhibiting the apoptosis of adipocytes induced by a hypoxic environment plays a crucial role in promoting the survival of fat grafts,^35^, ^36^ it is particularly important to have an in‐depth exploration of the underlying molecular mechanisms and to identify new molecules that work under hypoxic conditions. In the present study, we identified a novel secretory glycoprotein, LRG‐1, that could significantly increase the long‐term fat retention rate and inhibit the apoptosis of adipocytes in vivo. In addition, our in vitro experiment showed that LRG‐1 could suppress the apoptosis of ADSC under hypoxic conditions. Many studies on fat transplantation have reached the same conclusion as we,^37^, ^38^, ^39^ that is, inhibiting the apoptosis of fat grafts can increase their retention rate. However, they did not study the inhibition of ADSC apoptosis under hypoxic conditions. Our research on LRG‐1 inhibiting apoptosis of ADSC under hypoxic culture may better mimic the hypoxic conditions in transplanted fat. Moreover, since necrosis was also found in ADSCs under hypoxic conditions and autophagy was found inhibited by LRG‐1 in previous studies,^40^, ^41^ we conducted further experiments and found that LRG‐1 had no significant effects on proliferation, autophagy and necroptosis which better confirms the mechanism that LRG‐1 rescues ADSCs by inhibiting apoptosis but not by inhibiting other types of cell death or promoting cell proliferation. However, damaged or dead cells produce endogenous adjuvant substances called damage‐associated molecular patterns (DAMPs), which can induce local inflammation by recruiting immune cells such as macrophages.^42^ The inflammatory factors and reactive oxygen species released by the inflammatory reaction can also cause the death of transplanted fat cells. The immunohistochemical results of our study showed that LRG‐1 application significantly suppressed the number of F4/80^+^ macrophages in fat grafts. Whether LRG‐1 directly inhibits macrophage infiltration or inhibits inflammation by inhibiting apoptosis remains to be further studied.

Furthermore, by analysing high‐throughput data and carrying out related experiments, we demonstrated that RAB31, a member of the Ras oncogene family, mediated the inhibitory effect of LRG‐1 on ADSC apoptosis under hypoxic conditions. Our in vitro experiment demonstrated that the addition of LRG‐1 promoted the expression of RAB31 and decreased the expression of the apoptotic proteins Bax and cleaved caspase‐3. In addition, we found that when RAB31 was knocked down by specific siRNA, the decreasing trend of Bax and cleaved caspase‐3 recovered. Consistent with our research, other studies also showed that RAB31 is closely related to apoptosis inhibition. Researchers found that silencing RAB31 enhanced apoptosis and affected the expression of cell cycle and apoptotic proteins.^28^ Another study also found that Rab31 could inhibit U87 and SiHa cell apoptosis and decrease the expression of the apoptotic proteins Bax and PIG3.^27^ However, there are few studies on how RAB31 is regulated. Very few reports indicate that miR‐30a‐3p and miR‐30c‐2‐3p are upstream regulators of RAB31.^28^, ^43^ In this study, we found that the addition of LRG‐1 increased the expression of RAB31 under hypoxia, but hypoxia itself did not change the expression of RAB31. As a member of the Ras oncogene family, the high expression of RAB31 has always been thought to be closely related to the enhancement of tumour invasiveness,^44^, ^45^, ^46^, ^47^ the decrease in apoptosis and the poor prognosis of patients. Therefore, although the high expression of RAB31 can inhibit ADSC apoptosis under hypoxia, whether it is tumorigenic needs further experiments. Our in vivo results showed that the injection of LRG‐1 increased the expression levels of RAB31 in the transplanted fat. When RAB31 was knocked down by shRNA, the fat grafts were smaller and lighter than the fat grafts of the AAV‐shCtrl injection group. It is gratifying that during our in vivo experimental period, we did not observe the tumorigenicity of RAB31 expression.

Interestingly, a paradoxical role of LRG‐1 on cell apoptosis has been found in some previous studies,^41^, ^48^, ^49^ a recent review has discussed the functions of LRG‐1 by combining with TGFβ‐1 and cyt c.^50^ When Cyt c is bound to LRG1, cell survival is promoted, at least for lymphocytes in vitro. LRG‐1 appears to induce apoptosis through canonical TGF‐β1 signalling and cell survival through non‐canonical TGF‐β1 signalling. As a result, the expressions of Cyt c and TGF‐β1 vary in different physiological and pathological process of different cells, the paradoxical roles of LRG‐1 in apoptosis can be understood and the mechanisms of Cyt c or TGF‐β1 in fat graft survival can be studied in the future.

Besides, in terms of clinical translation, the application strategy of LRG‐1 should be considered. Given the relatively long and frequent injections, our current method of injecting LRG‐1 every other day for 2 weeks after fat grafting is not feasible. Therefore, we consider the co‐injection of the fat and LRG‐1 after liposuction and an additional injection of LRG‐1 3 days after fat grafting. We fully consider the effectiveness of this strategy since ADSCs experiencing a period of severe hypoxia within 7 days.^15^, ^40^ However, these are only our current assumptions; the details of application need to be further explored.

Moreover, the side effects in application of LRG‐1 are taken into consideration. Given that LRG‐1 functions in a variety of biological processes and acts as an emerging player in disease pathogenesis. We propose oncogenicity^51^, ^52^, ^53^, ^54^, ^55^ and inflammation as two possible side effects^56^, ^57^, ^58^ due to previous studies. The fact that there is currently no sign of tumour formation in our nude mice injected with LRG‐1 slightly lessened our concerns and the negative effect of LRG‐1 on inflammatory infiltration suggested a positive role in the survival of transplanted fat, which needs to be further studied. We also evaluate the triglyceride and free fatty acid (FFA) concentrations in plasma (Figure S3A) and the expression level of ATGL and HSL in liver showed that LRG‐1 addition had no significant effect on lipolysis in liver (Figure S3B), which suggested that local injection of LRG‐1 had no effect on liver metabolism.

In conclusion, we showed here that LRG‐1 could promote the survival of transplanted fat and inhibit its apoptosis in vivo. LRG‐1 reduced ADSC apoptosis induced by hypoxic conditions in vitro. In addition, the mechanism underlying the LRG‐1 suppression of ADSC apoptosis induced by hypoxic conditions was mediated by RAB31 upregulation. These data represent a clinically relevant mechanism in which transplanted fat exists in a hypoxic environment. Accordingly, LRG‐1 is a promising therapeutic target in fat transplantation.

## CONFLICT OF INTEREST

The authors declare that they have no competing interests.

## AUTHOR CONTRIBUTIONS

**Qingfeng Li:** Conceptualization (lead); Writing – review & editing (equal). **Chia‐kang Ho:** Data curation (equal); Formal analysis (equal); Investigation (equal); Project administration (equal). **Danning Zheng:** Data curation (equal); Funding acquisition (equal); Investigation (equal); Resources (equal); Software (equal). **Jiaming Sun:** Formal analysis (equal); Investigation (equal); Methodology (equal); Software (equal). **Dongsheng Wen:** Methodology (equal); Software (equal). **Shan Wu:** Resources (equal); Software (equal). **Li Yu:** Conceptualization (equal); Validation (equal); Writing – review & editing (equal). **Ya Gao:** Conceptualization (equal); Investigation (equal); Supervision (equal); Writing – review & editing (equal). **Yifan Zhang:** Conceptualization (equal); Funding acquisition (equal); Supervision (equal); Writing – review & editing (equal).

## Supporting information

Fig S1‐S6

## Data Availability

Datasets related to this article hosted at [Gene Expression Omnibus] (GSE122527) can be found at [https://www.ncbi.nlm.nih.gov/geo/query/acc.cgi?acc=GSE122527].
