# Supplementary material for: LRG‐1 promotes fat graft survival through the RAB31‐mediated inhibition of hypoxia‐induced apoptosis
Source: J Cell Mol Med. 2022 Mar 23;26(11):3153–68. doi: 10.1111/jcmm.17280 (PMC9170820; doi:10.1111/jcmm.17280)
Supplement: Supplementary file 1 — Fig S1‐S6 [file JCMM-26-3153-s001.docx]

Supplementary Fig. 1 LRG-1 facilitated the long-term survival of transplanted fat at month 12. (A) Representative image of macroscopic views of fat grafts in the control and LRG-1-treated groups at month 12. (B) Representative image of macroscopic views of calcification and oil cyst in fat grafts in different groups at month 12. (C) Images of H&E-stained sections of the calcification and oil cyst in fat grafts at month 12.


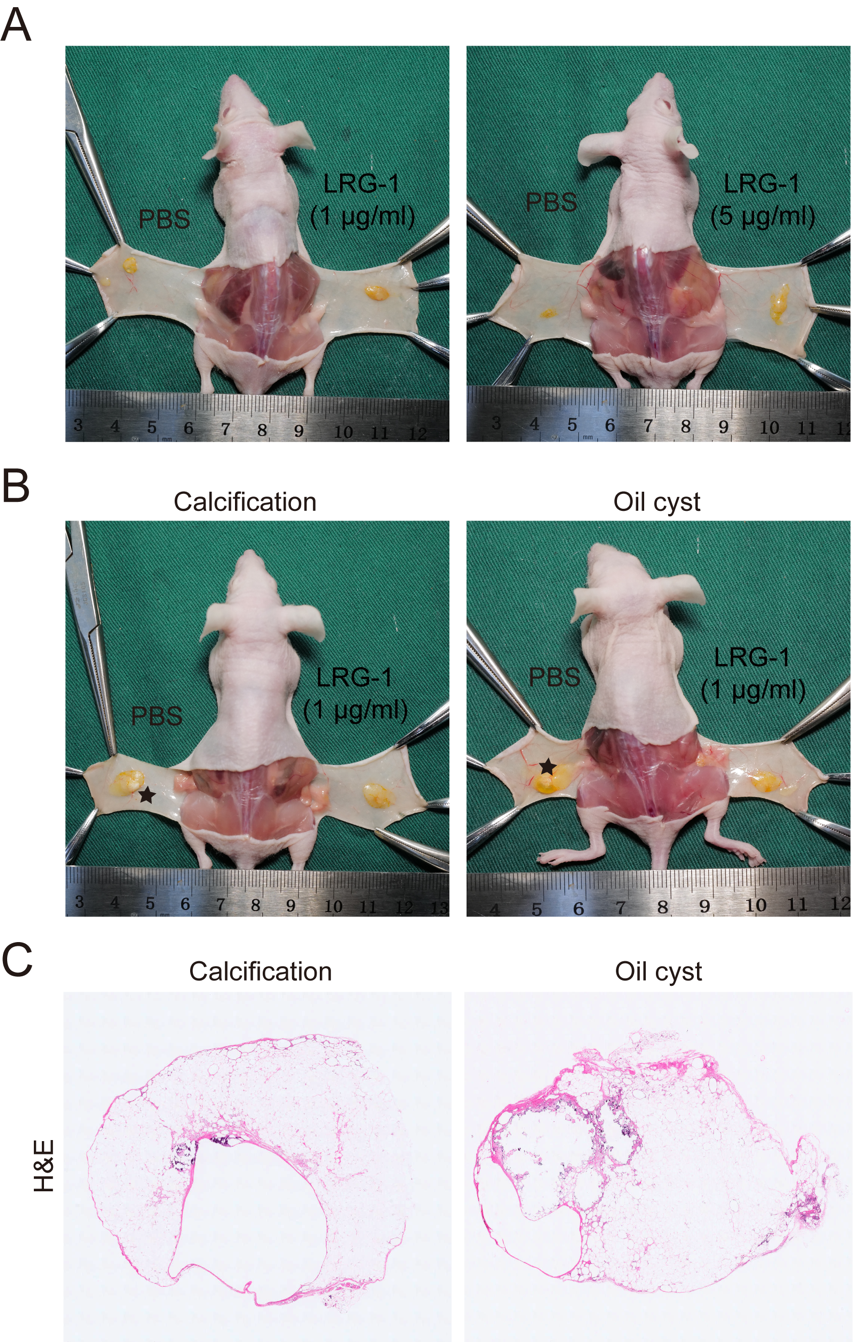


Supplementary Fig. 2 Immunohistochemistry staining of F4/80. Images and quantitative analysis of immunohistochemistry staining of F4/80 in fat grafts in the PBS injection and LRG-1 injection groups. (Scale bar = 200/100 μm). Data are presented as the mean ± SD. (n = 8 biologically independent animals) ***P < 0.001


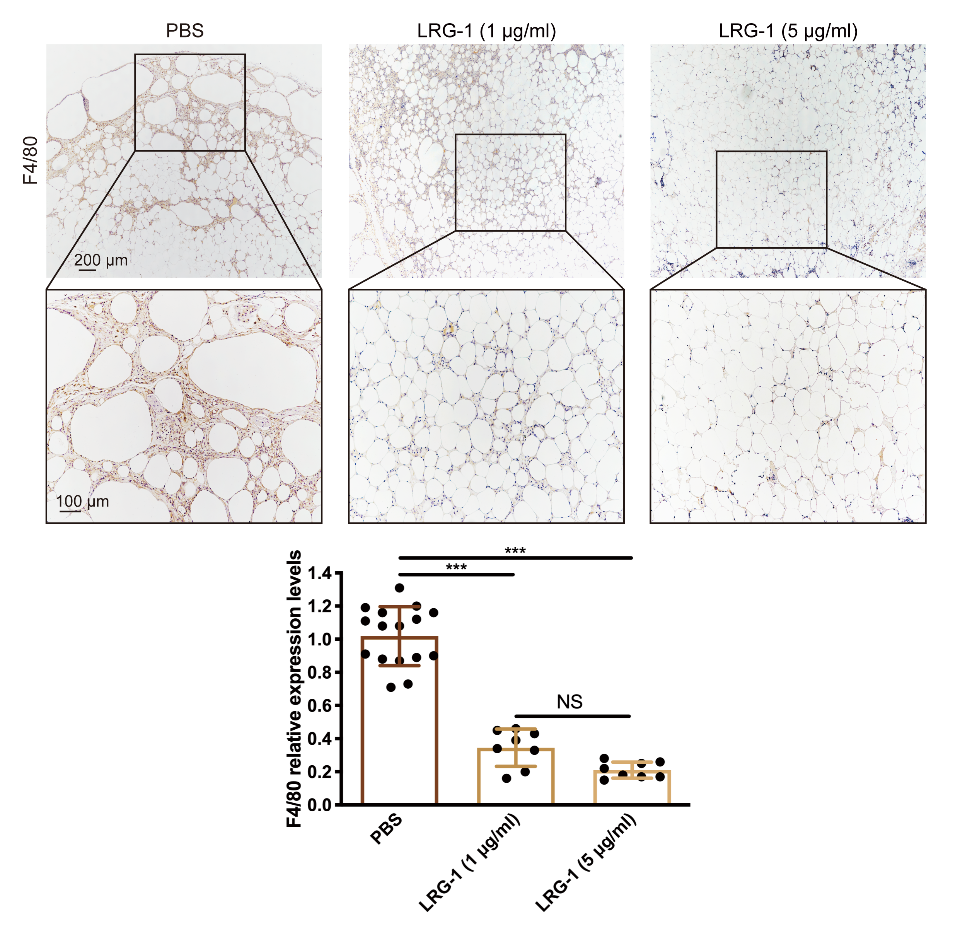


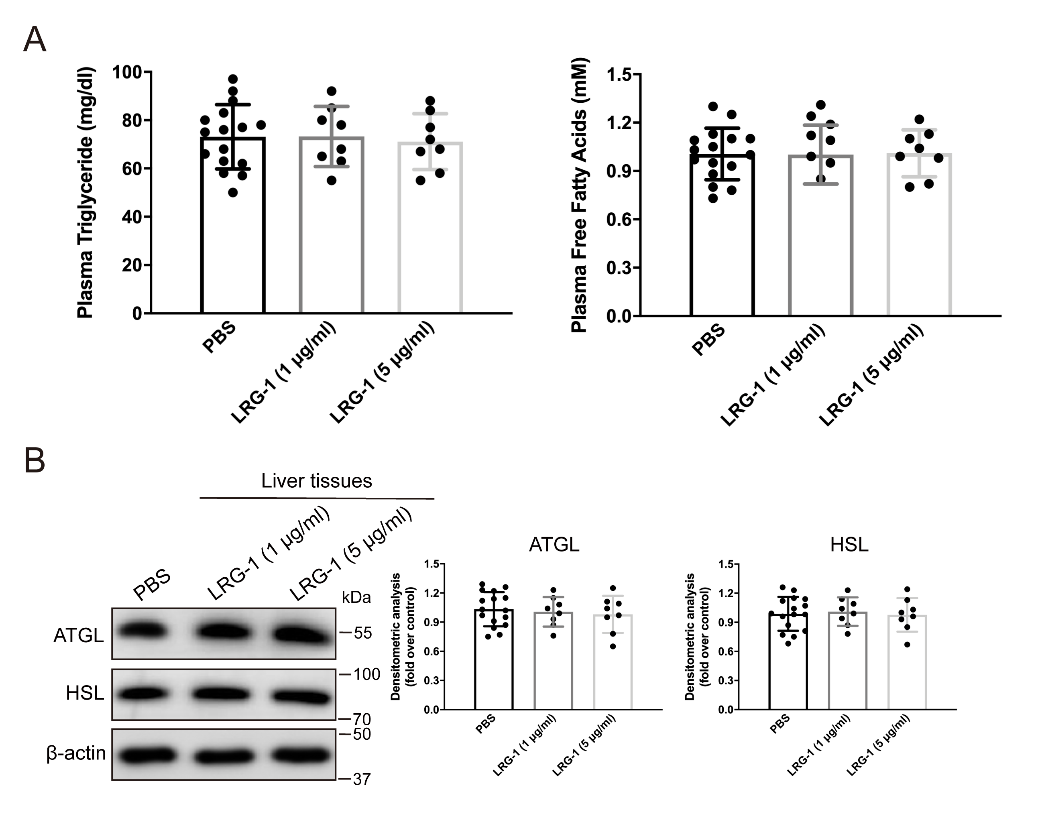
Supplementary Fig. 3 LRG-1 had no effect on lipolysis in plasma and liver. (A) The level of triglyceride and free fatty acid in plasma was detected in the control and LRG-1-treated groups. (B) Western blot analysis of ATGL and HSL in liver tissues of fat grafted mice in different groups. Data are presented as the mean ± SEM. (n=8 biologically independent animals)


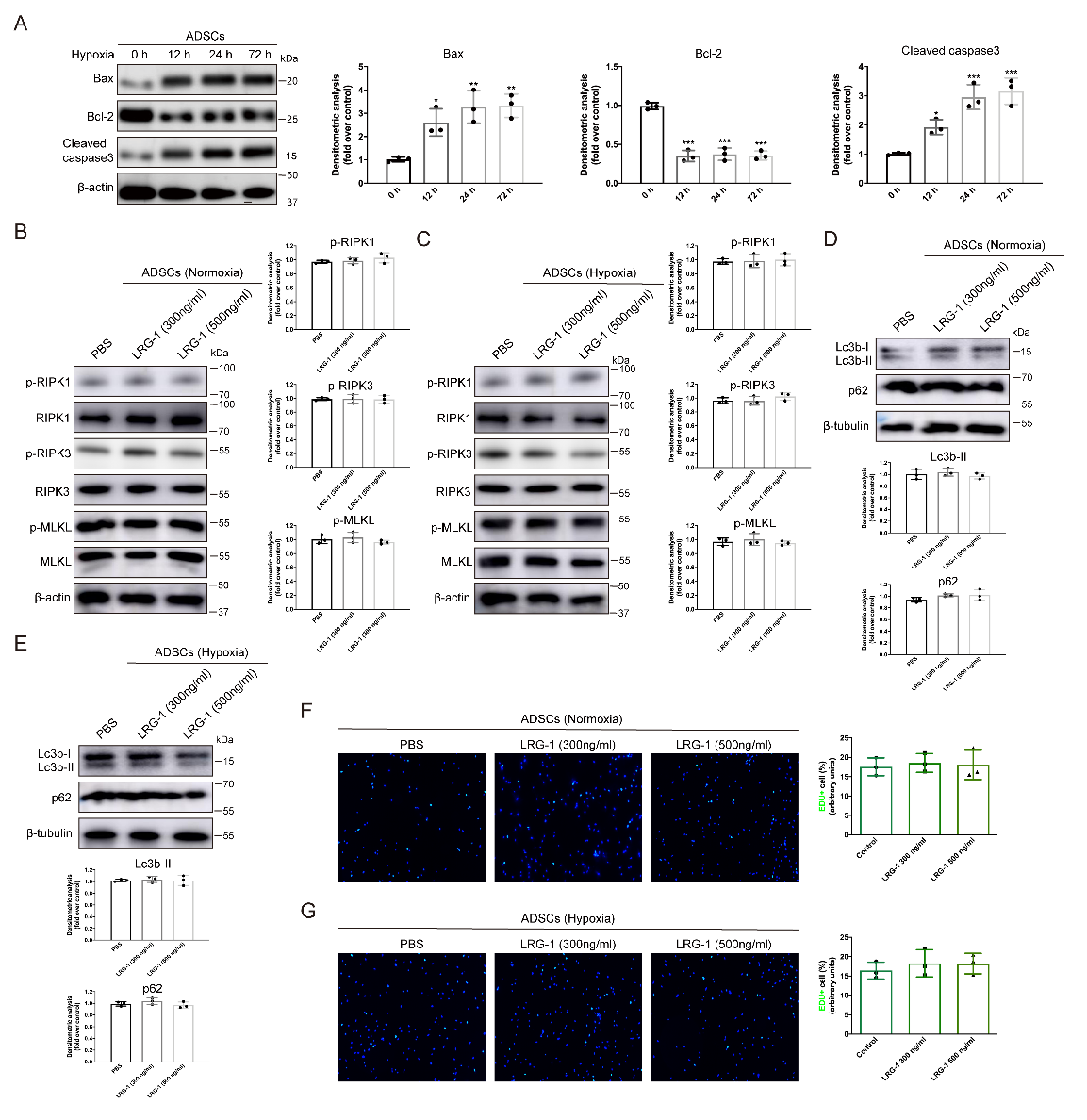
Supplementary Fig. 4 Hypoxia induced apoptosis of h-ADSC and LRG-1 had no effect on autophagy, necroptosis and proliferation of h-ADSC. (A) Western blot analysis of Bax, Bcl-2 and cleaved caspase-3 in h-ADSC cultured under hypoxia (1% O2) at different time points. (B) Western blot analysis of p-RIK1, p-RIPK3, p-MLKL in h-ADSC after treatment with PBS or LRG-1 (300 ng/ml or 500 ng/ml) for 48 hours under normal oxygen (21% O_2_). (C)Western blot analysis of p-RIK1, p-RIPK3, p-MLKL in h-ADSC after treatment with PBS or LRG-1 for 48 hours under hypoxia. (D)Western blot analysis of Lc3b and p62 in h-ADSC after treatment with PBS or LRG-1 for 48 hours under normal oxygen. (E) Western blot analysis of Lc3b and p62 in h-ADSC after treatment with PBS or LRG-1 for 48 hours under hypoxia. (F) EdU (green) proliferation assay was performed in h-ADSC after treatment with PBS or LRG-1 for 48 hours under normal oxygen. DAPI-stained nuclei are blue. (G) EdU proliferation assay was performed in h-ADSC after treatment with PBS or LRG-1 for 48 hours under normal oxygen. Data are presented as the mean ± SEM. (n=3 independent experiments) **P* < 0.05, ***P* < 0.01, ****P* < 0.001


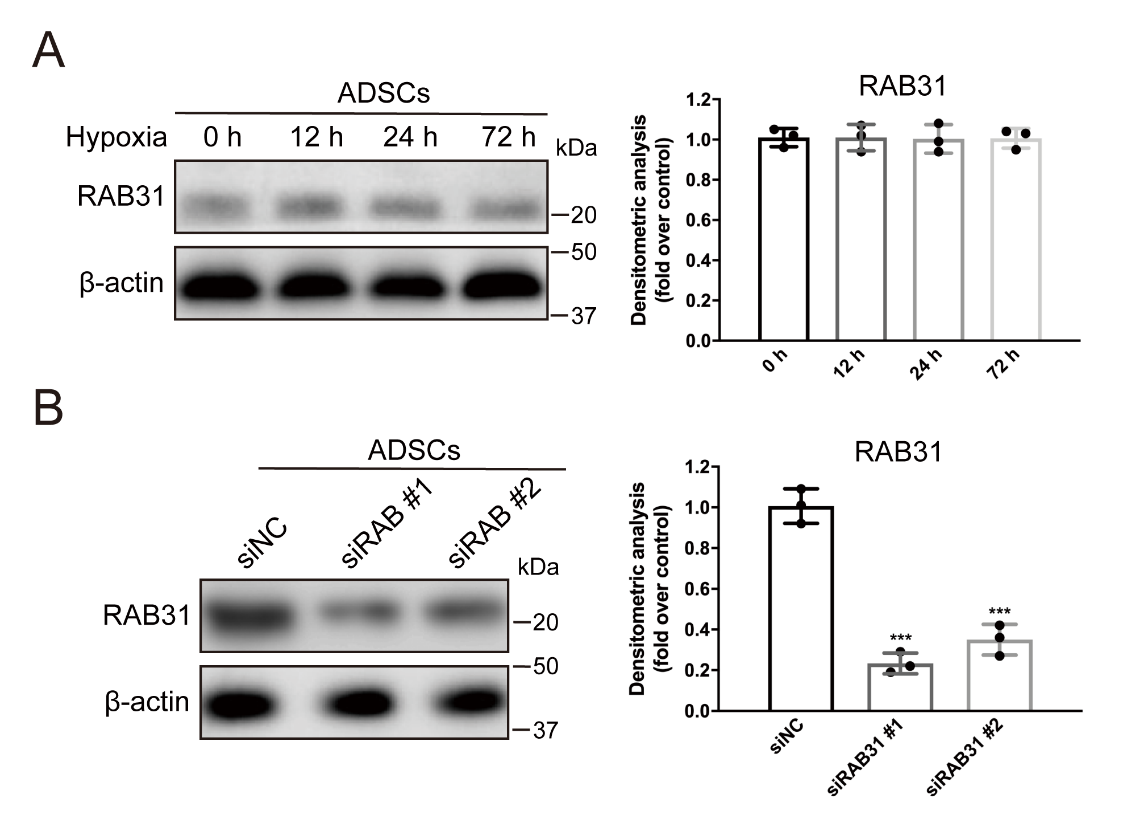
Supplementary Fig. 5 Hypoxia did not induce the expression of RAB31. (A) Western blot analysis of RAB31 in h-ADSC cultured under hypoxia (1% O2) at different time points. (B) Protein levels of RAB31 in h-ADSC transfected with si-RAB31 and siNC. Data are presented as the mean ± SEM. (n=3 independent experiments). ***P < 0.001


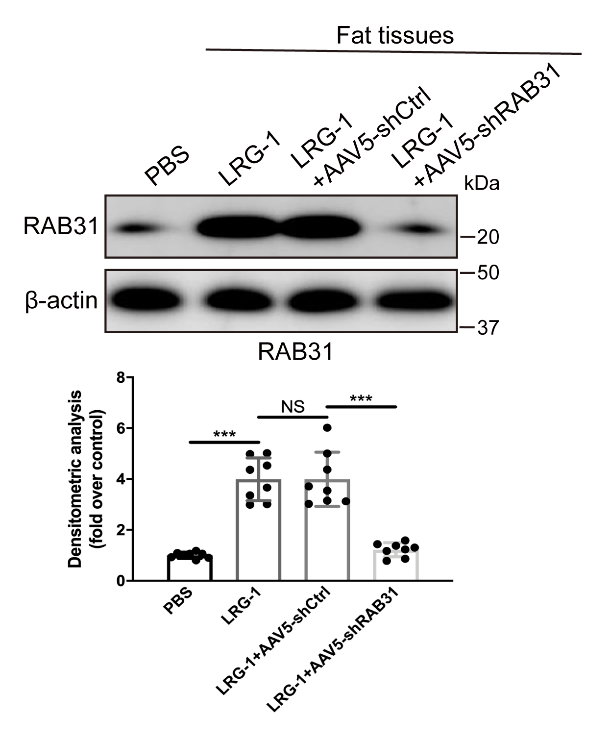
Supplementary Fig. 6 Identification of AAV5-shRAB31 efficiency in fat grafts. Protein levels of RAB31 in four different groups of fat grafts. Data are presented as the mean ± SD. (n = 8 biologically independent animals) ***P < 0.001
